# Process optimization for the supercritical carbondioxide extraction of lycopene from ripe grapefruit (*Citrus paradisi*) endocarp

**DOI:** 10.1038/s41598-021-89772-6

**Published:** 2021-05-13

**Authors:** Supriya Priyadarsani, Avinash Singh Patel, Abhijit Kar, Sukanta Dash

**Affiliations:** 1 Division of Food Science and Postharvest Technology, ICAR-Indian Agricultural Research Institute, New Delhi, 110 012 India; 2 Department of Food Science and Human Nutrition, School of Food and Agriculture, University of Maine, Orono, ME 04469 USA; 3 Division of Design and Experiments, ICAR – Indian Agricultural Statistics Research Institute, New Delhi, 110 012 India

**Keywords:** Drug discovery, Plant sciences

## Abstract

In this study, an underutilized citrus family fruit named grapefruit was explored for the extraction of lycopene using supercritical carbon dioxide (CO_2_) extraction technique. An experimental design was developed using response surface methodology to investigate the effect of supercritical carbon dioxide (CO_2_) operating parameter viz., pressure, temperature, CO_2_ flow rate, and extraction time on the extraction yield of lycopene yield from grapefruit. A total of 30 sets of experiments were conducted with six central points. The statistical model indicated that extraction pressure and extraction time individually, and their interaction, significantly affected the lycopene yield. The central composite design showed that the polynomial regression models developed were in agreement with the experimental results, with R^2^ of 0.9885. The optimum conditions for extraction of lycopene from grapefruit were 305 bar pressure, 35 g/min CO_2_ flow rate, 135 min of extraction time, and 70 °C temperature.

## Introduction

The overwhelming evidence on the reduced risk of dreadful diseases by consuming vegetable- and fruit-rich diets has triggered humankind to search for new biological resources. These natural resources contain several bioactive phytochemicals, which fight against many human diseases and are used as food preservatives and colorants in the food industry. Many researchers have found that natural food colors are mostly inherited from anthocyanin, carotenoids, chlorophyll, betalains, iridoids, phycobiliproteins, etc.^1–4^. Among them, carotenoids have gained more importance and attracted extensive investigation due to its dominating antioxidant properties^5–7^. Around 600 types of carotenoids have been identified on earth. About 40 of these carotenoids have been found in the human diet. However, only 14 types of carotenoids can be absorbed in the human digestive tract, primarily lycopene, α- & β- carotene, zeaxanthin, and lutein others^8–10^. Lycopene is one of the most significant carotenoids due to its dominant antioxidant properties, compared to β-carotene and the others. Lycopene is a red-colored compound and has the identical chemical and molecular structure to the β-carotene. It is a long-chain hydrocarbon (–CH) compound that contains 40 carbons with alternating 13 double bonds and eight isoprene units^1^. Several studies have investigated the free radical invasion potential of the lycopene at the cellular membrane surface. Studies have shown that it triggers the primary defence mechanism of the human body against several chronic diseases and plays a central role in mitigating oxidative stress as it lessens the inducible nitric oxide synthase activation^1,8,11^.

The top five lycopene sources among fruits and vegetables are watermelon, pink guava, papaya, tomatoes, and grapefruit, containing the lycopene content of up to 72, 53, 53, 42 34 µg/g (wet weight) respectively^1^. These products, except grapefruit, are commercially used in our daily lives. Nonetheless, although it contains a relatively fair amount of lycopene, grapefruit is limited by its higher acidity level and significantly bitter taste^12^. Grapefruit (*Citrus paradisi*) is a subtropical citrus fruit, also known as *“fruit from the paradise”* because of its health imparting and disease-preventing properties. It contains several phytochemicals such as lycopene, β-carotene, ascorbic acid, vitamin A and naringin. The presence of naringin causes the consumer’s unacceptability of the grapefruit as it causes a strong bitter taste, which results in very limited or no utilization^12,13^. Very few attempts have been made for the value addition to grapefruit, such as solvent extraction as well as ultrasound assisted extraction of lycopene^14,15^, and also by using petroleum ether as extraction solvent^16^ for successful extraction of this bioactive pigment called lycopene. Apart from this, oil extraction and isolation of polysaccharides has also been carried out from grapefruit^17^. However, it is necessary to carry out a sustainable and food-grade extraction of lycopene from grapefruit to commercialize as an antioxidant and natural colorant into the food system.

The extraction of lycopene from fruits and vegetables is done using a solvent extraction method, which is the most prevalent and commercial method used in the natural colorant industries. Using standard non-polar solvents like hexane, tetrahydrofuran, and chloroform, is non-sustainable and unsuitable for human consumption^15,18^. The solvent extraction process has several disadvantages: toxicity, disposal of solvent, a large quantity of organic solvent needed for extraction, high extraction temperatures, prolonged extraction time, less extraction efficiency, and dilution of the extract^19,20^. Moreover, higher extraction temperatures and prolonged extraction time lead to significant lycopene degradation, resulting in the isomerization of stable *trans*-isomer to a relatively unstable *cis*-isomer^21,22^.

These setbacks of the solvent extraction process necessitate the development of safer and cleaner methods of extraction of lycopene. One such technique is the supercritical fluid extraction (SFE), which utilizes supercritical fluids (the solvent) to separate the desired component (extractant) from the intricate food matrix^19,23^. The extraction of biotic compounds using a supercritical fluid emerges to be a green technology and produces the purest, cleanest, and safest product among all known extraction techniques available today. Although costlier, it is preferred because of its higher extraction efficiency and absence of residual solvent in the extracted material. Besides, it allows working at moderate temperatures, which invariably reduces degradation caused by thermal effect. Light and oxygen’s negligible presence significantly enhances stability and prevents oxidation reactions^19,20^. It is also non-toxic and smoothly gets separated from the extract. Carbon dioxide is generally the preferred solvent in this technique because it converts into the supercritical stage at 31 °C under a pressure of 74 bars^19,23^. Several extraction parameters such as extraction temperature, pressure, time, CO_2_ flow rate, co-solvent ratio (i.e., ethanol), among others, can be controlled to enhance the extraction efficiency and retain the quality of extracts maximally. So, it is essential to understand that the extraction conditions that maximally influence polyphenolics compounds’ stability and oxidative degradation^19,24^.

Therefore, this study was investigated to understand the effect of the supercritical extraction parameter such as temperature, pressure, time, and CO_2_ flow rate on the extraction yield of lycopene from the freeze-dried grapefruit powder. The central composite rotatable design (CCRD) of response surface methodology (RSM) was used to design the experiment. The extraction yield of the lycopene was measured on supercritical fluid chromatography.

## Materials and methods

### Materials and reagents

The ripe grapefruit was procured from the Division of Fruits and Horticultural Technology, IARI, New Delhi, India. It was harvested at a stage when the fruit exhibited 2/3rd yellow color. Lycopene was purchased from the Sigma Aldrich (St. Louis, MO, USA). Liquid CO_2_ with the purity of over 99.5% was purchased from the Amit Labs, New Delhi, India. Other solvents used in quantifying extracted lycopene were purchased from Merck Life Science Pvt. Ltd. Mumbai, India.

### Supercritical carbon dioxide extraction

The grapefruits were peeled, diced into small pieces, and lyophilized at − 52 °C for four days under dark condition. The lyophilized grapefruits were ground into powder using hammer mill and passed through a 250 µm size SI sieve to restrict the particle size. The powder was packed in LDPE zip lock bag and stored at − 20 °C for supercritical fluid extraction.

**Figure 1:**
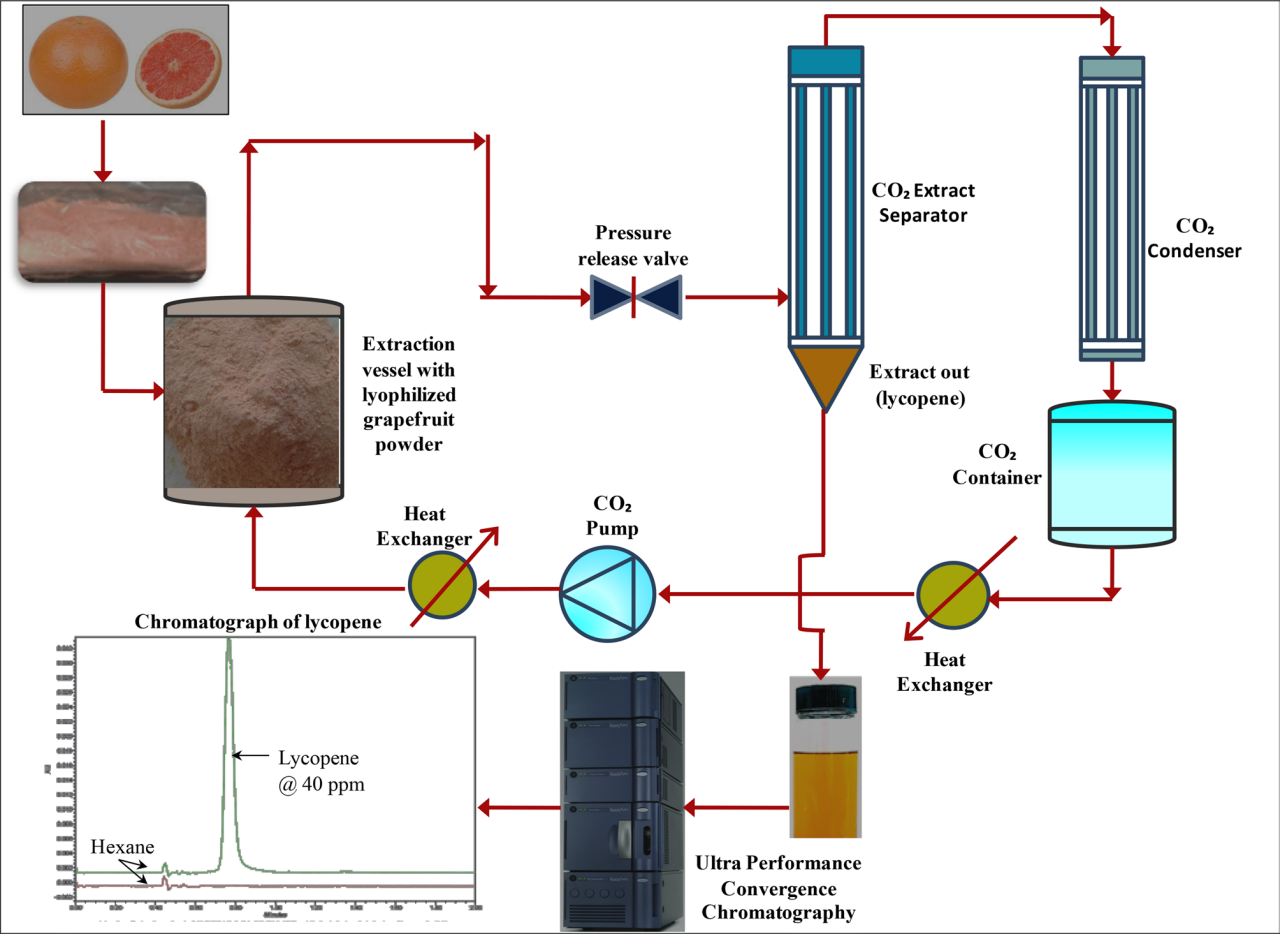
Schematic of the supercritical carbon dioxide extraction and characterization of lycopene from grapefruit.

A 100 g of the powdered grapefruit was loaded into the extraction vessel of the supercritical carbon dioxide extraction system, Thar Technologies, USA (Model No. 7100). The operating parameters i.e. extraction temperature, pressure, CO_2_, and time were controlled using the operating software SuperChrom SFC Suite, Thar Technologies, USA (v5.9 version). ethanol @ 5% along with supercritical CO_2_ @ 95% was used as extraction solvent. Ethanol was used as a co-solvent with CO_2_ to enhance extraction yield^23,25^. A schematic of the supercritical carbon dioxide extraction of lycopene is given in Fig. 1. The extracted lycopene was collected in amber colored bottle and stored at − 20 °C before further quantitative study.

### Quantification of lycopene

**Figure 2:**
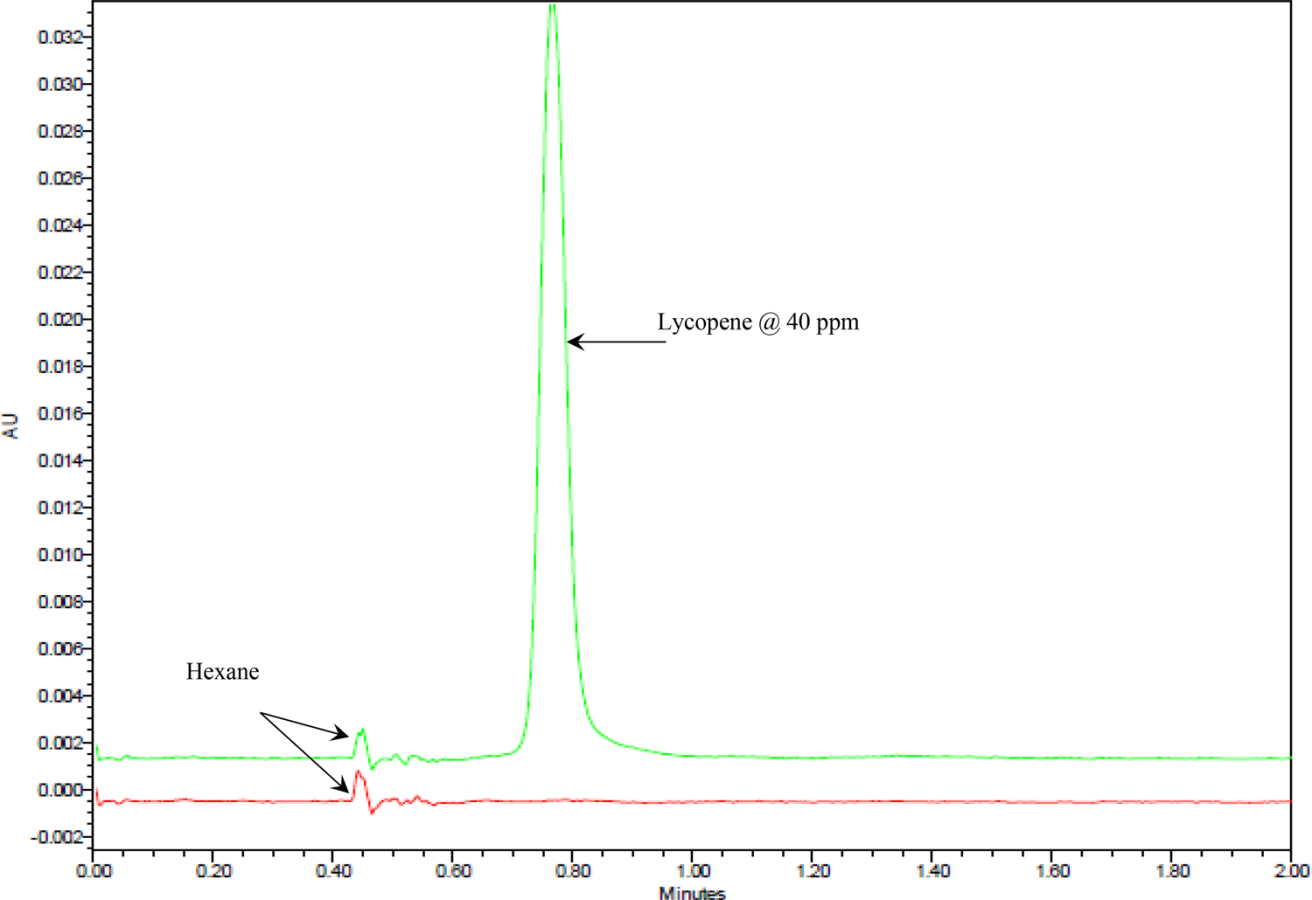
Chromatograms for blank (hexane) and hexane spiked with 40 ppm lycopene standard at using BEH 2 EP 2.1 × 150 mm, 5 µm column at optimized experimental conditions.

The lycopene’s extraction yield was measured on supercritical fluid chromatography as per the protocol suggested by Runco et al.^26^, with some modification. The extracted lycopene was vacuum dried and dissolved into hexane. Dissolved lycopene was filtered through 0.22 µm of pore size PVDF membrane (Millipore Ltd., Germany), and 1 mL was loaded into a 1.5 mL amber-colored vial. Quantification was performed on UPC^2^Acquity System (Waters Technologies, USA) equipped with a BEH 2-EP column (2.1 × 150 mm × 5 µm) and a photodiode array detector. The absorbance was recorded at 452 nm. The mobile phase consisted of CO_2_ and methanol of 85:15 (v/v) run under isocratic conditions. The chromatographs of the lycopene and n-hexane are given in Fig. 2.

### Experimental design and statistical analysis

The experiment was designed using a 2^4^ full factorial central composite design (CCD) with five levels of temperature (50, 60, 70, 80 & 90 °C); pressure (150, 225, 300, 375 & 450 bars); CO2 flow rate (15, 25, 35, 45 & 55 g/min); and extraction time (45, 90, 135, 180 & 225 min). Thirty sets of experiments were conducted (Table 1). Evaluation of the yield (response variable, Y) was done using a full second-order polynomial model of the design considering the yield to be a function of independent variables (x) and their interactions (Eq. 1)

1 $$ \begin{aligned} Y & = \beta_{0} + \beta_{1} X_{1} + \beta_{2 } X_{2} + \beta_{3} X_{3} + \beta_{4} X_{4} + \beta_{11} X_{1}^{2} \\ & \quad + \beta_{22} X_{2}^{2} + \beta_{33} X_{3}^{2} + \beta_{44} X_{4}^{2} + \beta_{12} X_{1} X_{2} + \beta_{13} X_{1} X_{3} \\ & \quad + \beta_{14} X_{1} X_{4} + \beta_{23} X_{2} X_{3} + \beta_{24} X_{2} X_{4} + \beta_{34} X_{3} \\ \end{aligned} $$

where Y is the response (yield), β_0_ is the constant coefficient, β_i_, β_j_ and β_ij_ are the linear, quadratic, and interaction coefficients respectively, and x_i_, x_j_are the coded values of independent variables.

**Table 1:** Experimental combinations.

No. of Exp	Pressure (bar)	Flow rate (g/min)	Time (min)	Temperature (°C)
1	225	25	90	60
2	225	45	180	60
3	375	25	180	60
4	225	25	180	80
5	225	45	90	80
6	375	25	90	80
7	375	45	90	60
8	375	45	180	80
9	300	35	135	70
10	300	35	135	70
11	300	35	135	70
12	300	35	135	70
13	300	35	135	70
14	300	35	135	70
15	225	25	180	60
16	375	25	90	60
17	375	25	90	80
18	225	25	90	80
19	375	45	180	60
20	225	45	180	80
21	375	25	180	80
22	375	45	90	80
23	300	35	135	90
24	300	35	135	50
25	450	35	135	70
26	150	35	135	70
27	300	55	135	70
28	300	15	135	70
29	300	35	45	70
30	300	45	225	70

The design point for the different process variables was obtained using the PROC RSREG procedure of the SAS software (version 9.3, SAS Institute, Cary, NC, USA) with no coding option. It was used for analysis by Response Surface Methodology (RSM) of the experimental data obtained. Five replicates at the center of the design were used to estimate a pure error sum of squares.

## Results and discussion

Recovery of lycopene from grapefruit at different experimental runs varied between 13.4% and 77.2% (Table 1). The recovery percentages were calculated by evaluating the lycopene content in the fruit powder before extraction, recovered lycopene using SFE and the lycopene content in the residual powder. Analysis of the data indicated that the lycopene yield from grapefruit could be adequately described using a second order quadratic model as follows:

2 $$ \begin{aligned} {\text{Y}} & = 47.72512 - 0.08{\text{X}}_{1} + 0.56{\text{X}}_{2} + 0.13{\text{X}}_{3} + 0.14{\text{X}}_{4} - 0.0011{\text{X}}_{1} {\text{X}}_{2} \\ & \quad + 0.002{\text{X}}_{1} {\text{X}}_{3} + 0.0028{\text{X}}_{1} {\text{X}}_{4} - 0.000476{\text{X}}_{2} {\text{X}}_{3} + 0.0048538{\text{X}}_{2} {\text{X}}_{4} \\ & \quad + 0.00399{\text{X}}_{3} {\text{X}}_{4} - 0.0011{\text{X}}_{1}^{2} - 0.0303{\text{X}}_{2}^{2} - 0.001594{\text{X}}_{3}^{2} - 0.0632{\text{X}}_{4}^{2} \\ \end{aligned} $$

where Y is the lycopene recovery (%), X_1_, X_2_, X_3_ and X_4_ represents pressure, flow rate, time and temperature respectively.

Validity of the statistical model is generally adjudged by the lack of fit to check adequacy of the model (Table 2(A)). Significance of the model and a non-significance of the lack of fit at five percent level of significance indicated that the developed model for lycopene yield prediction from grapefruit was a good fit. An R^2^ value of 0.99 reinforces the same.

**Table 2:** Analysis of variance (ANOVA) (A) response surface quadratic model for the lycopene yield from grapefruit and (B) Independent variables and their interactions for lycopene yield from grapefruit.

Source	Degrees of freedom	Sum of squares	Mean square	F ratio	Prob > F
(A)					
Model	14	6352.0824	453.720	2.6514	0.0355
Lack of Fit	9	2463.8977	273.766	15.9536	0.2093
Pure Error	6	102.9612	17.160		
Total	29	8918.9414	R^2^ = 0.99		

Source	Degree of freedom	Sum of squares	F ratio	Prob > F	
(B)					
Pressure (bar)	1	977.7444	5.7137	0.0304	
Flowrate (g/min)	1	741.9673	4.3358	0.0549	
Time (min)	1	853.2729	4.9863	0.0412	
Temperature (C)	1	47.9752	0.2804	0.6042	
Pressure × pressure	1	1230.3897	7.1901	0.0171	
Pressure × flow rate	1	10.7287	0.0627	0.8057	
Flow rate × flow rate	1	250.6241	1.4646	0.2449	
Pressure × time	1	1175.4394	6.8689	0.0193	
Flowrate × time	1	0.6657	0.0039	0.9511	
Time × time	1	284.3462	1.6616	0.2169	
Pressure × temperature	1	63.6863	0.3722	0.5510	
Flowrate × temperature	1	3.3712	0.0197	0.8902	
Time × temperature	1	46.9175	0.2742	0.6082	
Temp × temperature	1	1089.45	6.3664	0.0234	

ANOVA (Table 2(B)) of the effect of linear, quadratic and interaction effects of the variables indicated that the pressure (X1) and time (X3) individually as well as their interaction effect significantly affected the lycopene yield. However, in its quadratic term, pressure and temperature were found to have significant effect on lycopene yield. All other linear, quadratic and interaction effects were found to be non-significant at 5% level of confidence (*P* < 0.05).

**Figure 3:**
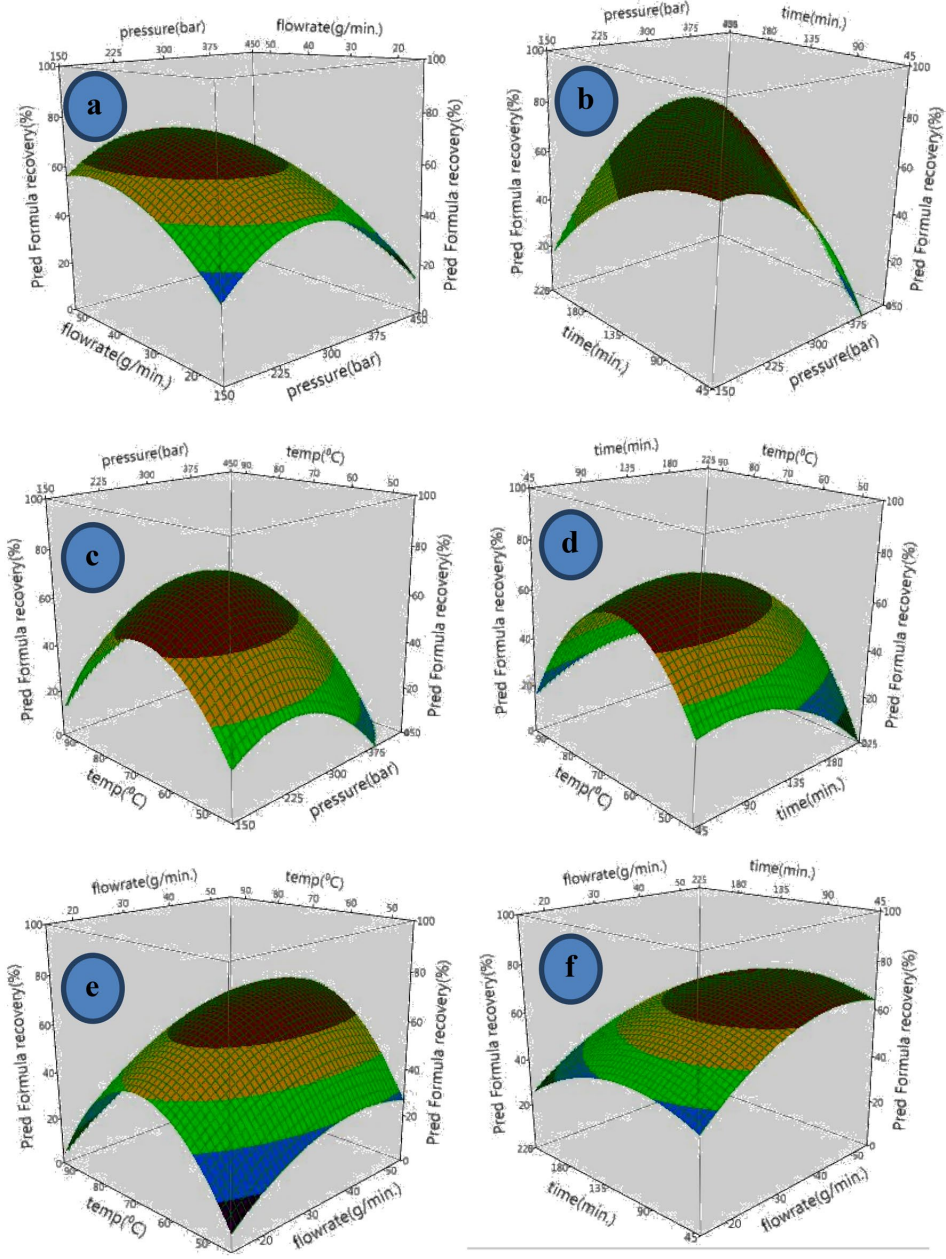
Response surface plot showing effects of two independent variables on lycopene yield from grapefruit while the remaining were kept at the central point (Pressure—300 bar; Flow rate—35 g/min; Time—135 min; and Temperature—70 °C). [SAS (r) Proprietary Software 9.4 (TS1M1), Copyright (c) 2002–2012 by SAS Institute Inc., Cary, NC, USA].

Effect of independent variables on lycopene extraction was done by analysing the response surface graphs plotted between any two independent variables keeping the third at the central point (Fig. 3a–f).

### Effect of independent variables of lycopene extraction

#### Pressure and time

With an increase in pressure from 150 to 300 bars, it has been observed that lycopene yield increased at a given flow rate of the supercritical CO_2_and further reduced with increase in pressure up to 450 bars. The rate of increase however gradually reduces with the increase in the flow rate (Fig. 3a). Besides, lycopene yield increased with increase in the flow rate of supercritical CO_2_ from 15 to 45 g/min and almost got stabilized with further increase in flow rate up to 55 g/min. This increase may be attributed to the fact that the increased pressure enhanced solvent density of the supercritical CO_2_ resulting in enhanced solubility of lycopene as it has been reported by some researchers too^25,31^. Nevertheless, beyond a certain limit, pressure plays a negative role by decreasing the diffusion ability of the solvent because of the enhanced compaction of the samples at higher pressure leading to channelling of the supercritical CO_2_ around it rather than diffusing through it^25,30,33^.

An increase in pressure decreases lycopene yield significantly at lower extraction time up to about 100 min. The trend, however, reverses to a significant increase with the increase in pressure.The increase of lycopene yield with pressure could have been due to increased solvent density of the supercritical CO_2_ resulting in enhanced solubility of lycopene^25,31^. The response surfaces indicated the significant (*p* < 0.05) effect of both pressure and time individually as well as in conjugation over lycopene yield, which reinforces the significance of these factors found in ANOVA (Table 2). The time of extraction is an indicative measure of the availability of the quantity of supercritical CO_2_ for the extraction process. The completion of the extraction is adversely affected if the available supercritical CO_2_ is a limiting factor. Nevertheless, beyond a certain point, any increase in extraction time could have a detrimental effect because of the overpowering of other factors such as temperature^24,27,28^. There is a significant (*p* < 0.05) role in both positive and negative terms in the extraction of lycopene from the matrix. The positive effect involves enhancing the extraction rate by solvent densification, whereas the negative effect of sample compaction is higher. Hence, an appropriate balance between the two parameters is of utmost importance for maximizing lycopene yields^23,25^.

#### Temperature

Temperature plays a vital role in any extraction process involving supercritical CO_2_. Increase in lycopene yield is observed with the increase in extraction temperature upto80°C, irrespective of pressure and time of extraction. This is because higher temperatures are known to enhance the solubility of solute, thereby increasing mass transfer of solute in the matrix^29–31^. However, increase in extraction temperature beyond 80 °C causes a significant reduction in the lycopene yieldfrom around 80% to about 30%. This can be explained by the loss of balance between the supercritical CO_2_ density and solute vapour pressure^19,23,31^ and lycopene degradation because of isomerization^24,27^.

#### Flow rate

An increase in the flow rate of the supercritical CO_2_ from 15 to 45 g/min was found to increase lycopene yield in the entire range of temperatures considered in the study. This can be attributed to enhanced dissolution rate of lycopene into the supercritical CO_2_ because of universal concentration gradient phenomenon^24,25^. However, a further increase in the CO_2_ flow rate beyond 45 g/min significantly (*p* > 0.05) reduced the lycopene extraction yield. The reduction in extraction yields may have been because of reduced interaction between the solute (grapefruit powder) and solvent (ethanol + supercritical CO_2_)^28,30^. The reduced interaction is because of the solvent quickly passing around attributed to the solvent the sample matrix at a higher flow rate rather than defusing through it. This description is strengthened by the surface plot between extraction and the flow rate of supercritical CO_2_ (Fig. 3). With the increase in extraction time and the flow rate of supercritical CO_2_, it was observed that the lycopene yields significantly increased. This increase is primarily because the solvent’s improved dwell time with the solutes triggering the solvent’s penetration into the grapefruit sample matrix which enables maximization of extraction of lycopene^32,33^.

Taking into account the interaction effect of the independent parameters, up to an extraction time of 100 min, the lycopene yield decreases with increase in pressure. The trend however, reverses to a significant increase with the increase in pressures (Fig. 3b). The surface in fact clearly indicates the significant effect of both pressure and time individually as well as in conjugation over lycopene yieldwhich reinforces the significance of these factors found in ANOVA (Table 2). The time of extraction is an indicative measure of the amount of supercritical CO_2_ available for the extraction process. In case the available supercritical CO_2_ is a limiting factor, the completeness of extraction is adversely affected. However, increase in time period beyond a point where the available supercritical CO_2_ suffices the completeness of extraction, could lead to detrimental effect because of other controlling parameters like temperature^24,27,28^. Pressure level as described earlier could either play a significant role in aiding extraction by solvent densification or limit it because of sample compaction. Hence, an appropriate balance between the two is essential for maximization of lycopene yields.

### Optimization of extraction condition

Second order response surface was fitted to the responses (lycopene extraction) obtained in all the thirty experimental runs and stationary point was obtained through canonical analysis using SAS software. The nature of stationary point was evaluated and found to be the point of maxima and therefore considered the optima. At the optimum conditions i.e. a combination of 305 bars of pressure, 70 °C temperature, flow rate of 35 g/min of supercritical CO_2_, and 135 min. of extraction time, 93% extraction efficiency of lycopene was achieved. In order to validate the same five additional experimental runs were conducted using the optimum conditions. The experimental yield obtained was 91.03 ± 1.86%indicating a good agreement between the predicted and observed extraction yields. This clearly established the authenticity of the statistical model in predicting lycopene extraction yield at any given experimental combination of independent variables considered for the study.

## Conclusion

The results indicated the possibility of successfully achievingupto 93% extraction of lycopene from the grapefruit matrix using the optimum combinations of independent variables considered for the study. Statistical analysis revealed a non-significant effect of temperature, carbon dioxide (CO_2_), and their interaction on the extraction yield of the lycopene. Whereas, pressure, extraction time, and their interaction, had a significant (p < 0.05) effect on lycopene extraction. Second-order polynomial showed high regression coefficients and a well-fitted model to the obtained experimental data.

## Certificate

Certified that the use of plants parts in the present study complies with international, national and/or institutional guidelines.
